# Magnetic record of Mio-Pliocene red clay and Quaternary loess-paleosol sequence in the Chinese Loess Plateau

**DOI:** 10.1016/j.dib.2017.11.059

**Published:** 2017-11-21

**Authors:** Yougui Song

**Affiliations:** State Key Laboratory of Loess and Quaternary Geology, Institute of Earth Environment, Chinese Academy of Sciences, Xi'an 710061, China

**Keywords:** Rock magnetic record, Late Miocene and Pliocene red clay, Quaternary loess-paleosol sequence, Chinese Loess Plateau

## Abstract

This article presents magnetic data of a 300-m-thick Mio-Pliocene red clay and Quaternary loess-paleosol sequence near Chaona town in the Central Chinese Loess Plateau. Detailed magnetostratigraphy shows that the aeolian red clay began to accumulate at ca. 8.1 Ma. Here, we presented a high-resolution rock magnetic data at 20–40 cm intervals within 4.5–8 ka span per sample of this section, which has been published in Song et al. (2014) [1] and (2017) [2]. The dataset including the following magnetic parameters: mass magnetic susceptibility (*χ*), frequency-dependent susceptibility (*χ*_fd_), saturation magnetization (*M*_s_), saturation remanent magnetization (*M*_rs_), coercive force (*B*_c_), remanent coercivity (*B*_cr_), saturation isothermal remanent magnetization (SIRM) and S-ratio. Magnetic susceptibility and hysteresis parameters were measured at Lanzhou University and Kyoto University, respectively. This data provides a high-resolution rock magnetic evidences for understanding East Asia Monsoon change, Asian interior aridification and tectonic effect of the uplift of the Tibetan Plateau since middle Miocene period.

**Specifications Table**

**Table:** 

Subject area	*Geology*
More specific subject area	*Quaternary geology and paleoclimate*
Type of data	*graphs, figures*
How data was acquired	Bartington MS2 susceptometer, 2 G cryogenic magnetometer (Model 760); Alternating Gradient Magnetometer Micromag^TM^ 2900 model.
Data format	*Raw and analyzed.*
Experimental factors	*Samples dry completely in room temperature before measuring.*
Experimental features	Magnetic susceptibilities were measured using a Bartington MS2 susceptometer at frequencies of 470 Hz and 4700 Hz. The magnetic remanence was measured on an American DSM2 spinner magnetometer or 2 G cryogenic magnetometer (Model 760). Progressive alternating field demagnetization with 11–18 steps were carried out for most of the samples at 2--10 mT intervals to 70 mT, and progressive thermal demagnetization was done for some typical samples at 20--100 °C intervals in about 22 steps from a room temperature to 710 °C; and Magnetic hysteresis parameters were determined by an Alternating Gradient Magnetometer Micromag^TM^ 2900 model.
Data source location	35°06'N, 107°12'E; Chaona town, Lingtai County, Gansu Province, China
Data accessibility	*Data are within this article and related references*
Related research article	*Song et al. 2005; 2007; 2014;2017.*

**Value of the Data**

• Provide a high-resolution rock magnetic record of the last 8 Ma for local or global environmental changes comparison.
• It is helpful to understand the East Asia Monsoon revolution and Asian interior aridification.
• Provide some clues for understanding the uplift process of the Tibetan Plateau and its effects during late Cenozoic period.
• Useful to researchers and experts working in environmental magnetism, paleoclimate change, past global changes, Quaternary geology and other related fields.

## 1 Data

The Late Cenozoic red clay-loess-paleosol sequence in the Chinese Loess Plateau provides a high-resolution record of paleoclimatic and tectonic changes in Asian. Detailed magnetostratigraphy of a 300-m-thick late Miocene-Pliocene red clay and Quaternary loess-paleosol sequences near Chaona town in the central Chinese Loess Plateau shows that the red clay began to accumulate at ca. 8.1 Ma [3], [4]. Different time scales based on astronomically tuning [5] or independent untuning grain-size age models [1], [6] were established. During the past decade, grain size [7], the pollen [8], [9], carbonate [10], and heavy mineral [11], [12], [13] content, together with the magnetic susceptibility enhancement mechanism [14], [15], [16], and their palaeoenvironmental significance [17], [18], [19], have been investigated.

Here, we presented a high-resolution rock magnetic data at 20–40 cm intervals within 4.5–8 ka span per sample in this Section [1], [2]. The dataset including the following magnetic parameters: mass magnetic susceptibility (*χ*), frequency-dependent susceptibility (*χ*_fd_), hysteresis parameters including saturation magnetization (*M*_s_), saturation remanent magnetization (*M*_rs_), coercive force (*B*_c_) and remanent coercivity (*B*_cr_), Saturation isothermal remanent magnetization (SIRM) and *S*-ratio. Magnetic susceptibility (*χ*) and SIRM are function of magnetic mineralogy, concentration and granulometry. Paleosol layers developed under warm and wet interglacial periods have relatively higher both *χ* and *χ*_fd_ because of stronger pedogensis in the Chinese Loess Plateau, they have widely been employed as proxies of East Asian summer monsoon intensity. The shapes of the hysteresis loops may be an indicator of the degree of pedogenesis. *M*_rs_ and *M*_s_ are closely related to the type and content of magnetic minerals. Higher *M*_rs_ and *M*_s_ values indicate an increase in ferrimagnetic minerals, such as magnetite and maghemite, and lower values imply the absence of strongly ferrimagnetic minerals. The high coercivity (*B*_c_, *B*_cr_) may imply the presence of antiferromagnetic goethite and hematite. Rock magnetic data show that the loess layers are characterized by relatively high coercivity (*B*_c_ and *B*_cr_), lower magnetic susceptibility (*χ*, *χ*_fd_), and the paleosol layers are characterized by relatively high *χ*, *χ*_fd_ and SIRM. S-ratio is a good indicator of the proportion of magnetite to hematite, higher S-ratio means more abundant concentration of magnetite than hematite.

Based on the magnetic data, we reconstructed the history of the East Asia monsoons during the last 8 Ma and its possible driving mechanism [1], [2], [6]. We also explored the middle Pleistocene climate transition in this section, which began at ~1.26 Ma and was completed by ~0.53 Ma [1]. Our work also indicated that the variations of both the East Asian Summer Monsoon and Winter Monsoon before 4.3 Ma were closely related to global cooling and that the intensified Summer Monsoon during the late Pliocene was primarily caused by tectonic events, including the gradual closure of the Panama Seaway and the uplift of the Tibetan Plateau, rather than by global cooling [2].

## 2 Experimental design, materials, and methods

### 2.1 Materials

The Chaona section is located at Zhengjiashizi Village (107°13´E, 35°02´N) about 5 km south from Chaona Town of Lingtai County, Gansu Province. The section is composed with 175 Quaternary loess-paleosol sequence and 125 late Miocene-Pliocene red clay sequence with a paleomagnetic age 8.1 Ma [3], [4]. More than 400 sets of oriented block samples at 0.2–2 m interval for paleomagnetic measurements and over 2600 discrete samples at 0.10–0.2 m interval for various proxies analyzes were collected [1], [2].

### 2.2 Magnetochronology

**Fig. 1:**
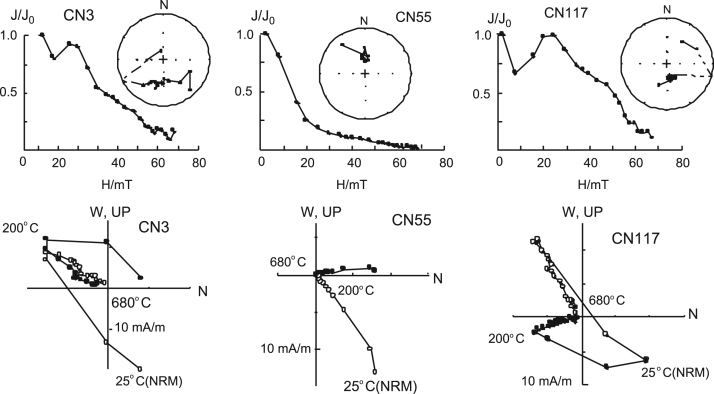
Progressive alternative (upper) and thermal (lower) demagnetization of some typical samples.

Each oriented bulk sample was split into three sets of parallel cubic specimens with a dimension of 2 × 2 × 2 cm for paleomagnetic analysis. The paleomagnetic measurement was performed in Lanzhou Geological Institute, and Geology and Geophysics Institute of the Chinese Academy of Sciences, and rechecked in the Paleomagnetism Laboratory of Kyoto University. The magnetic remanence was measured on an American DSM2 spinner magnetometer or 2 G cryogenic magnetometer. 11–18 steps of progressive alternating field demagnetization (AFD) were carried out for most of the samples at 2–10 mT intervals to 70 mT, and progressive thermal demagnetization (ThD) was done for some typical samples at 20–100 °C intervals in about 22 steps from a room temperature to 710 °C (Fig. 1).The magnetochronologic data indicates that bottom age of the section is around 8.1 Ma [3], [4].

### 2.3 Rock magnetic measurements

**Fig. 2:**
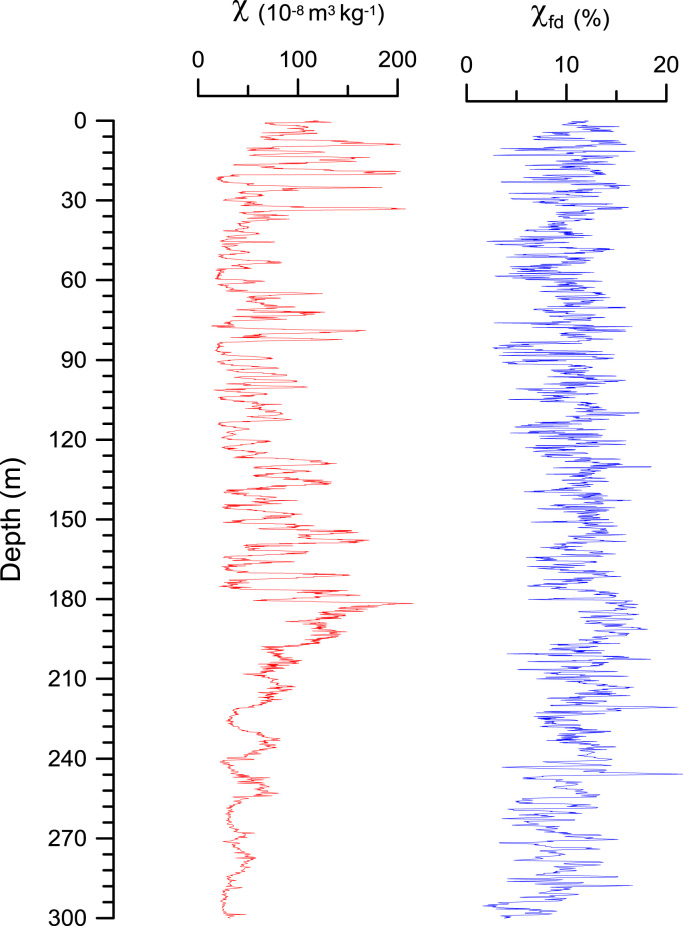
Magnetic susceptibility (*χ*) and frequency-dependence susceptibility (*χ*_fd_) variations with depth in the Chaona section in the central Chinese Loess Plateau.

Magnetic susceptibility and hysteresis parameters were measured at Lanzhou University and Kyoto University, respectively. Magnetic susceptibilities were measured on all air-dried samples using a Bartington MS2 susceptometer at frequencies of 470 Hz (i.e., *χ*_lf_) and 4700 Hz (i.e. *χ*_hf_), and *χ* [*χ* = (*χ*_lf_ + *χ*_hf_)/ 2], *χ*_fd_ [*χ*_fd_ = (*χ*_lf_−*χ*_hf_)/ *χ*_lf_*100] represent the absolute and relative behavior, respectively, of the frequency-dependent susceptibility (Fig. 2).

**Fig. 3:**
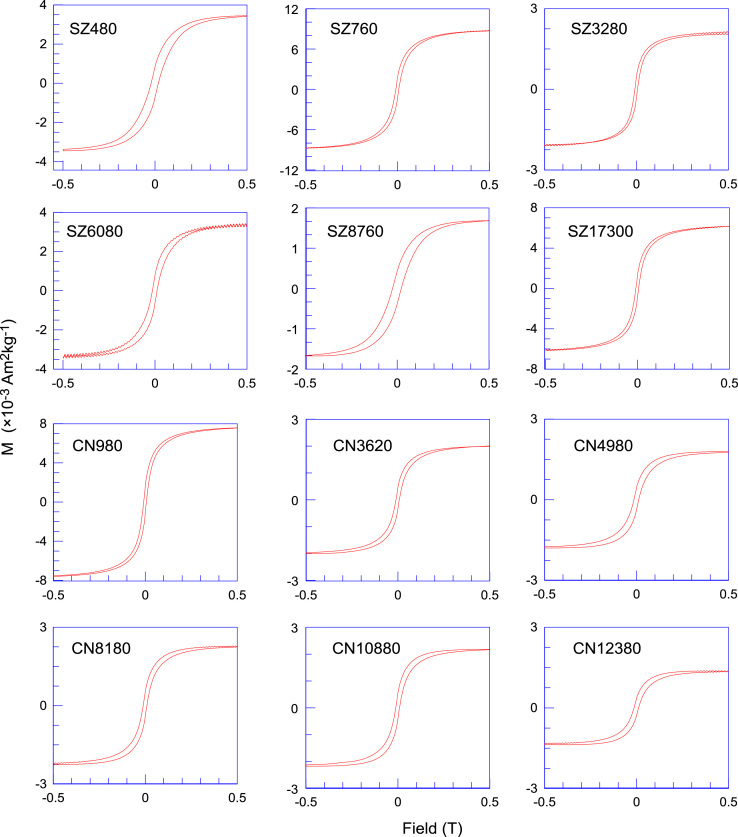
Representative magnetic hysteresis loops of loess-paleosols (SZ) and red clay (CN) samples from the Chaona section.

**Fig. 4:**
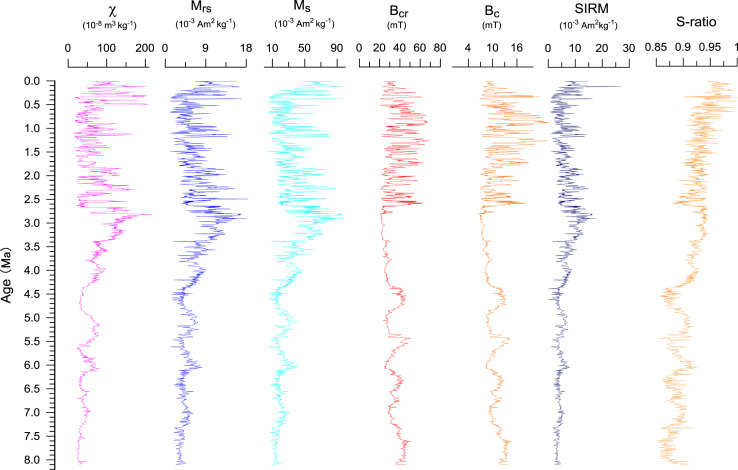
Time series of various rock magnetic parameters in the Chaona loess-paleosol and red clay sequences.

Magnetic hysteresis parameters (Fig. 3) were measured using an Alternating Gradient Magnetometer (AGM) Micromag^TM^ 2900 model at the paleomagnetism laboratory of Kyoto University, with a maximum field of 1.0 T and a 2 mT increment [1], [2]. Saturation magnetization (*M*_s_), saturation remanent magnetization (*M*_rs_) and coercive force (*B*_c_) were calculated after removal of the paramagnetic component. Remanent coercivity (*B*_cr_) was measured by applying a forward field of 1.0 T followed by the application of reverse fields of increasing strength and the change in remanence measured at each step. The data enable various interparametric ratios to be calculated using the IRM imparted at a high field (here 1.0 T) and the back IRM value at various reverse field strengths (e.g. −0.3 T). These parameters reflect variations in the coercivity spectrum of the magnetic mineral assemblage and therefore the mineralogy and grain size. The saturation isothermal remanent magnetization was determined at 1 T (SIRM), −0.1 T (IRM_−0.1 mT_) and −0.3 T (IRM_−0.3T_). S-ratio was calculated as S-ratio = [(-IRM_−0.3 T_/SIRM) + 1]/2. Based on paleomagnetic control points and independent untuned time scale, we established a high-resolution magnetic record of Mio-Pliocene red clay and Quaternary loess-paleosol sequence in this section (Fig. 4).
