# Supplementary material for: Magnetic record of Mio-Pliocene red clay and Quaternary loess-paleosol sequence in the Chinese Loess Plateau
Source: Data Brief. 2017 Nov 21;16:411–7. doi: 10.1016/j.dib.2017.11.059 (PMC5723265; doi:10.1016/j.dib.2017.11.059)
Supplement: Supplementary file 1 — Supplementary material [file mmc1.pdf]

Conflicts of Interest Statement

Manuscript title:

Magnetic record of Mio-Pliocene red clay and Quaternary loess-paleosol sequence in the Chinese Loess Plateau

The authors whose names are listed immediately below certify that they have NO affiliations with or involvement in any organization or entity with any financial interest (such as honoraria; educational grants; participation in speakers' bureaus; membership, employment, consultancies, stock ownership, or other equity interest; and expert testimony or patent-licensing arrangements), or non-financial interest (such as personal or professional relationships, affiliations, knowledge or beliefs) in the subject matter or materials discussed in this manuscript.

Author names:

Yougui Song

The authors whose names are listed immediately below report the following details of affiliation or involvement in an organization or entity with a financial or non-financial interest in the subject matter or materials discussed in this manuscript. Please specify the nature of the conflict on a separate sheet of paper if the space below is inadequate.

Author names:

Yougui Song

This statement is signed by all the authors to indicate agreement that the above information is true and correct (*a photocopy of this form may be used if there are more than 10 authors*):

Author's name (typed)	Author's signature	Date
Yougui Song	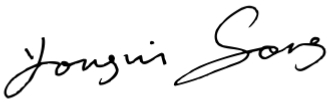	Nov 7， 2017
